# Identification of a novel prognosis-associated ceRNA network in lung adenocarcinoma via bioinformatics analysis

**DOI:** 10.1186/s12938-021-00952-x

**Published:** 2021-11-24

**Authors:** Yumiao Li, Xiaoxue Yu, Yuhao Zhang, Xiaofang Wang, Linshan Zhao, Dan Liu, Guofa Zhao, Xiangpeng Gao, Jiejun Fu, Aimin Zang, Youchao Jia

**Affiliations:** 1 Department of Medical Oncology, Affiliated Hospital of Hebei University, Hebei Key Laboratory of Cancer Radiotherapy and Chemotherapy, 212 Yuhua East Road, Baoding, 071000 Hebei People’s Republic of China; 2 College of Clinical Medicine, Hebei University, Hebei Key Laboratory of Cancer Radiotherapy and Chemotherapy, Baoding, 071000 Hebei People’s Republic of China; 3 Key Laboratory of Longevity and Aging-Related Diseases of Chinese Ministry of Education, Guangxi Medical University, Nanning, 530021 Guangxi People’s Republic of China

**Keywords:** Lung adenocarcinoma, LncRNAs, CeRNA, Biomarker, Prognosis

## Abstract

**Background:**

Lung adenocarcinoma (LUAD) is the most common subtype of nonsmall-cell lung cancer (NSCLC) and has a high incidence rate and mortality. The survival of LUAD patients has increased with the development of targeted therapeutics, but the prognosis of these patients is still poor. Long noncoding RNAs (lncRNAs) play an important role in the occurrence and development of LUAD. The purpose of this study was to identify novel abnormally regulated lncRNA–microRNA (miRNA)–messenger RNA (mRNA) competing endogenous RNA (ceRNA) networks that may suggest new therapeutic targets for LUAD or relate to LUAD prognosis.

**Methods:**

We used the SBC human ceRNA array V1.0 to screen for differentially expressed (DE) lncRNAs and mRNAs in four paired LUAD samples. Gene Ontology (GO) and Kyoto Encyclopedia of Genes and Genomes (KEGG) pathway analyses were performed to annotate the DE lncRNAs and mRNAs. R bioinformatics packages, The Cancer Genome Atlas (TCGA) LUAD database, and Kaplan–Meier (KM) survival analysis tools were used to validate the microarray data and construct the lncRNA–miRNA–mRNA ceRNA regulatory network. Then, quantitative real-time PCR (qRT-PCR) was used to validate the DE lncRNAs in 7 LUAD cell lines.

**Results:**

A total of 2819 DE lncRNAs and 2396 DE mRNAs (*P* < 0.05 and fold change ≥ 2 or ≤ 0.5) were identified in four paired LUAD tissue samples. In total, 255 of the DE lncRNAs were also identified in TCGA. The GO and KEGG analysis results suggested that the DE genes were most enriched in angiogenesis and cell proliferation, and were closely related to human cancers. Moreover, the differential expression of ENST00000609697, ENST00000602992, and NR_024321 was consistent with the microarray data, as determined by qRT-PCR validation in 7 LUAD cell lines; however, only ENST00000609697 was associated with the overall survival of LUAD patients (log-rank *P* = 0.029). Finally, through analysis of ENST00000609697 target genes, we identified the ENST00000609697–hsa-miR-6791-5p–RASL12 ceRNA network, which may play a tumor-suppressive role in LUAD.

**Conclusion:**

ENST00000609697 was abnormally expressed in LUAD. Furthermore, downregulation of ENST00000609697 and its target gene RASL12 was associated with poor prognosis in LUAD. The ENST00000609697–hsa-miR-6791-5p–RASL12 axis may play a tumor-suppressive role. These results suggest new potential prognostic and therapeutic biomarkers for LUAD.

**Supplementary Information:**

The online version contains supplementary material available at 10.1186/s12938-021-00952-x.

## Introduction

Lung cancer is the second most commonly diagnosed cancer worldwide, with 2.21 million new cases annually, and is the most common cause of cancer death (1.79 million deaths annually) [1]. Approximately 85% of lung cancer cases are nonsmall-cell lung cancer (NSCLC), and lung adenocarcinoma (LUAD) is currently the most common histological subtype of NSCLC [2–4]. Patients with late-stage disease at diagnosis have a poor prognosis. However, the occurrence, development, and prognosis of tumors are not only related to pathological type and clinical stage, but also closely related to abnormal gene expression in tumor cells [5] Early prevention and continuous improvements in targeted drugs support the clinical translation of the lung cancer treatment model, prolong the progression-free survival (PFS) and overall survival (OS) of patients, and improve their prognosis [6]. However, the prognosis of LUAD is still poor due to local recurrence or distant metastasis. Therefore, there is an urgent need to predict and explore more biomarkers for early diagnosis and therapeutic targets in LUAD.

Long noncoding RNAs (lncRNAs) are noncoding RNA (ncRNA) molecules of more than 200 nucleotides that are most commonly not translated into protein, but are crucial players in diverse cellular and physiological functions [7, 8]. Recently, with the development and application of high-throughput sequencing and gene chip technologies, researchers have found that lncRNAs play important roles in the occurrence and development of a variety of tumors [9]. LncRNAs that are abnormally expressed in tumor tissues can not only be used as specific tumor biomarkers for early diagnosis and prognosis, but also directly interact with DNA, messenger RNA (mRNA), or protein to regulate chromatin modification or structure or to affect gene transcription, splicing, and translation [10]. In general, lncRNAs can regulate a variety of physiological and pathological processes in tumor development, such as cell proliferation, differentiation, migration, and invasion; stem cell reprogramming; tumorigenesis; and drug resistance [11–15]. Competing endogenous RNAs (ceRNAs) are RNAs containing microRNA (miRNA) recognition elements (MREs) [16] that can regulate the expression of genes harboring the corresponding MRE or the expression of proteins by competitively binding to miRNAs [17]. LncRNAs can also act as ceRNAs and function within lncRNA–miRNA–mRNA ceRNA networks [17, 18]. Perturbation of ceRNA networks may affect diseases and explain disease processes; thereby, presenting opportunities for new therapies [17]. For instance, multiple studies have demonstrated that patients with various cancers with high HOTAIR expression exhibit higher lymphatic invasion and shorter survival times [19–24]. However, there is a need to discover new functional lncRNA–miRNA–mRNA ceRNA networks in LUAD. Therefore, research to identify more ceRNA networks related to LUAD diagnosis and prognosis is warranted.

In this study, we used gene chip technology to screen four paired LUAD tissue samples and analyzed and then predicted the multiple crucial functions of the identified DE lncRNAs and mRNAs. This analysis identified significantly more downregulated genes than upregulated genes. The data were also analyzed using R bioinformatics tools to construct the ceRNA regulatory network. Finally, we successfully screened the novel lncRNA ENST00000609697 and its target DE mRNA RASL12, which were negatively correlated with poor prognosis in LUAD. The results suggested that the ENST00000609697–hsa-miR-6791-5p–RASL12 ceRNA network may play a tumor-suppressive role and may contain new therapeutic targets and pathways related to LUAD survival.

## Results

### Identification of DE lncRNAs and mRNAs in LUAD and adjacent tissues

**Fig. 1:**
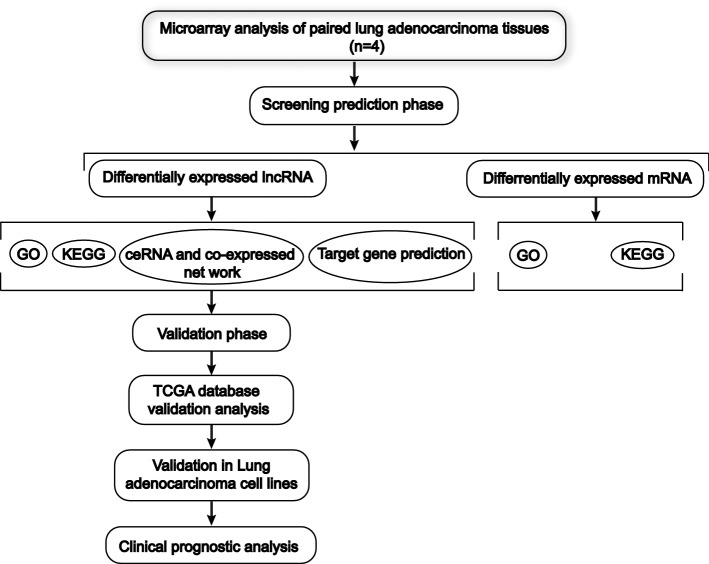
The flowchart of this study

**Fig. 2:**
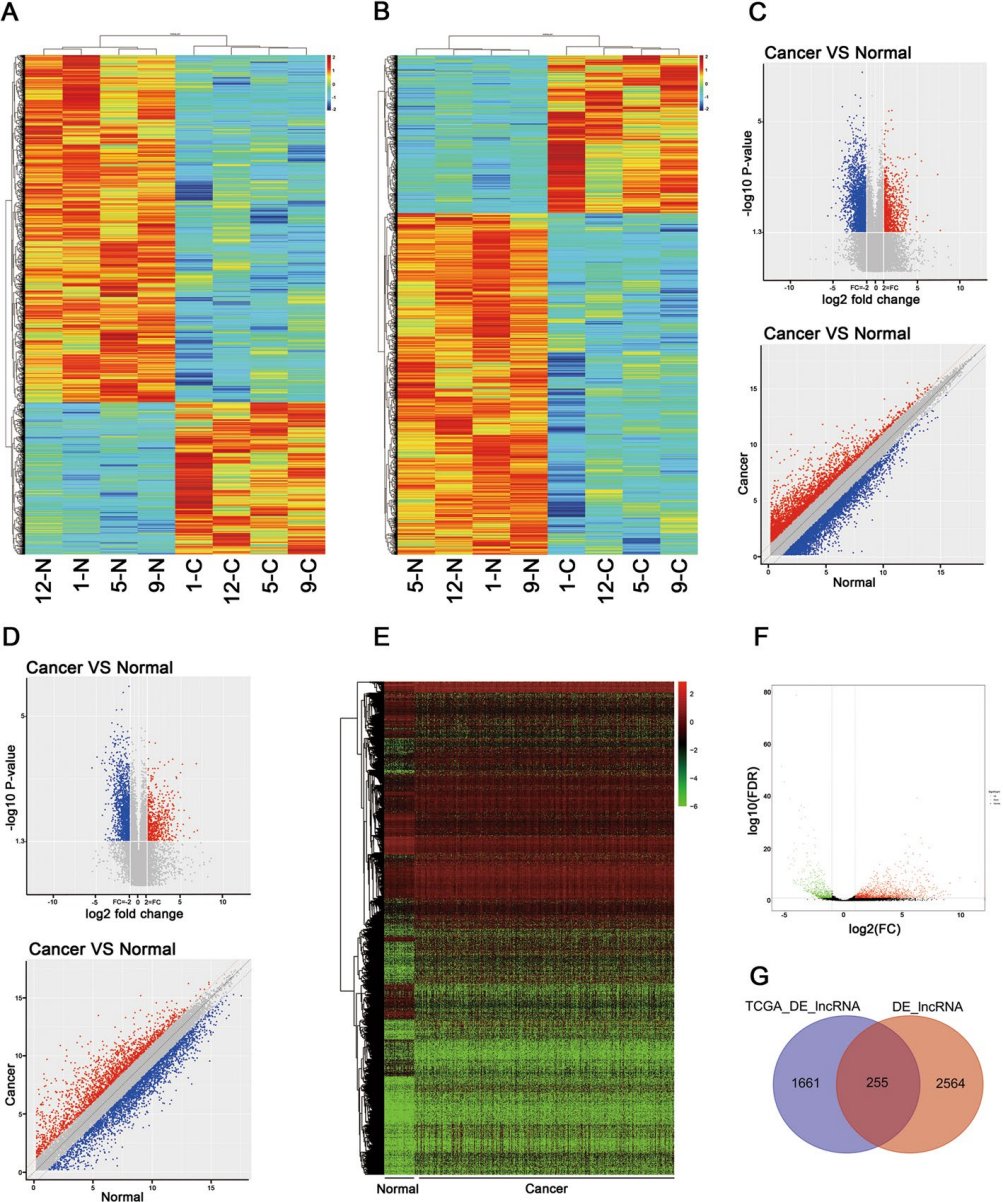
Screening of DE lncRNAs and mRNAs between LUAD tissues and normal tissues. **A** Heatmap of DE lncRNAs; **B** Heatmap of DE mRNAs; the *x* axis shows samples and the *y* axis shows DE genes. Red and blue represent upregulated and downregulated genes, respectively. **C** Scatter and volcano plots showing the expression profiles of DE lncRNAs based on the lncRNAs expression values detected by microarray. **D** Scatter and volcano plots showing expression profiles of DE mRNAs based on the expression values of mRNAs detected by microarray; |FC|≥ 2.0, *P* < 0.05. **E** Heatmap of DE lncRNAs in 512 LUAD tissue samples and 59 adjacent tissue samples. Green and red represent downregulated and upregulated lncRNAs, respectively. **F** Volcano plot of the 1271 upregulated lncRNAs and 645 downregulated lncRNAs that were screened (|log_2_FC|> 1, *P* < 0.05). **G** Venn diagram of DE lncRNAs identified by the TCGA and microarray analyses

In our study, microarray profiling was performed with four paired LUAD tissue samples (tumor and paracancerous tissues) to identify DE lncRNAs and mRNAs. An overview of this study is shown in Fig. 1. Overall, we identified 2819 lncRNAs and 2396 mRNAs with significant differential expression (*P* < 0.05 and fold change (FC) ≥ 2 or ≤ 0.5): 859 upregulated lncRNAs, 1960 downregulated lncRNAs, 757 upregulated mRNAs, and 1639 downregulated mRNAs. The hierarchical clustering heatmap showed the expression levels of the DE lncRNAs (Fig. 2A) and mRNAs (Fig. 2B) and the molecular signatures of these DE lncRNAs and mRNAs distinguish cancer from adjacent tissues. By volcano plot and scatter plot analyses evaluating the overall distribution of the two datasets, these DE RNAs were divided into up- and downregulated lncRNAs (Fig. 2C) and up- and downregulated mRNAs (Fig. 2D). The number of downregulated genes was significantly greater than that of upregulated genes. The top 20 significantly DE lncRNAs and mRNAs identified according to FC values are presented in Tables 1 and 2.

**Table 1:** Top-20 differentially expressed lncRNAs of lung adenocarcinoma and adjacent tissue samples

Accession	*P* values	FC(abs)	Regulation		Chr	GeneSymbol
ENST00000423781	0.045138791	154.273527	Up	chr7		AC004870.4
lnc-SYT16-1:1	0.008110801	62.1498876	Up	chr14		–
lnc-TSPAN13-2:1	0.000027973	34.927184	Up	chr7		–
ENST00000431027	0.007829215	26.7528941	Up	chr1		RP3-340N1.2
NR_046533	0.000809301	25.2378211	Up	chr13		CLDN10-AS1
lnc-BCKDHB-4:1	0.002525077	18.4268214	Up	chr6		–
ENST00000605886	0.04382729	16.6142073	Up	chr1		RP11-284F21.10
NR_125404	0.023786704	15.962481	Up	chr3		LOC100505920
lnc-USP26-3:1	0.003316762	15.7760352	Up	chrX		–
lnc-BCKDHB-6:1	0.006763185	13.5382121	Up	chr6		–
lnc-NSRP1-2:2	0.000161724	27.71072711	Down	chr17		–
ENST00000480831	0.001737636	15.74807358	Down	chr3		ADAMTS9-AS1
ENST00000432452	0.001497719	14.60031376	Down	chr10		RP11-464C19.3
lnc-GTDC1-15:1	0.000686192	14.54798755	Down	chr2		–
ENST00000443224	0.000787963	14.36781879	Down	chr10		RP11-371A19.2
lnc-ZPLD1-2:2	0.004414129	14.01720412	Down	chr3		–
ENST00000507525	0.000368778	13.3686819	Down	chr4		RP13-577H12.2
lnc-TRAPPC5-1:1	0.001126285	12.74950409	Down	chr19		–
NR_003928	0.012408513	12.17016938	Down	chr1		CHIAP2
ENST00000624132	0.015263542	11.88176057	Down	chr9		RP11-205K6.3

**Table 2:** Top-20 differentially expressed mRNA of lung adenocarcinoma and adjacent tissue samples

Accession	*P* values	FC(abs)	Regulation	Chr	GeneSymbol
NM_173076	0.00025142	112.2153919	Up	chr2	ABCA12
NM_003695	0.02604819	93.23154863	Up	chr8	LY6D
NM_001032280	0.01004802	63.89111709	Up	chr6	TFAP2A
NM_032899	0.00605935	41.28293568	Up	chr8	FAM83A
NM_001199042	0.03422378	40.11209326	Up	chr15	STRA6
NM_001164431	0.01571357	38.51455003	Up	chr20	ARHGAP40
NM_025153	0.00026241	35.86487577	Up	chr5	ATP10B
NM_001080407	0.02410958	30.83360008	Up	chr11	GLB1L3
NM_001251830	0.00046426	30.81333114	Up	chr4	SPP1
NM_001077188	0.00251909	30.572041	Up	chrX	HS6ST2
NM_012391	0.00602412	26.86301237	Up	chr6	SPDEF
NM_001045	0.00033657	45.9038257	Down	chr17	SLC6A4
NM_000261	0.000161133	30.0889688	Down	chr1	MYOC
NM_001114133	0.00024818	20.6970509	Down	chr10	SYNPO2L
NM_203451	0.000202297	19.3511029	Down	chr13	SERTM1
NM_001332	0.000243926	16.3150335	Down	chr5	CTNND2
NM_153370	0.000374892	15.9720912	Down	chr6	PI16
NM_021146	0.005748437	15.6408757	Down	chr1	ANGPTL7
NM_000575	0.000118471	14.9645892	Down	chr2	IL1A
NM_001278236	0.003914533	14.8213111	Down	chr11	PTPN5
NM_032961	0.000080895	14.6704759	Down	chr4	PCDH10

### Validation of the DE lncRNAs via the Cancer Genome Atlas (TCGA) database

To verify the microarray data in a large cohort of clinical samples, we downloaded the TCGA LUAD database, which contains both gene expression and patient survival data for the screened cohort, and obtained 573 samples (including 514 LUAD tissue samples and 59 adjacent tissue samples). The clinical patient information is presented in Table 3. As shown in the hierarchical clustering heatmap (Fig. 2E) and the volcano plot (Fig. 2F), 1916 DE lncRNAs (|log_2_FC|> 1, *P* < 0.05); namely, 1271 upregulated and 645 downregulated lncRNAs, were identified. As shown in the Venn diagram (Fig. 2G), the intersection of the 2819 DE lncRNAs identified by microarray analysis with the 1916 DE lncRNAs identified by TCGA database analysis contained 255 overlapping DE lncRNAs (Additional file 1: 255 overlapping genes).

**Table 3:** The clinicopathological characteristics of LUAD samples downloaded from TCGA database

Clinicopathological characteristics	Patients (*N* = 514)	
	*N*	%
Age		
< 68	280	54.4
≥ 68	224	43.6
Gender		
Male	240	46.7
Female	274	53.3
Pathologic staging		
Stage I	279	54.3
Stage II	122	23.7
Stage III	78	15.2
Stage V	26	2.5
Pathologic T		
T1	172	33.5
T2	277	53.9
T3	46	4.4
T4	18	1.7
Tx	4	< 0.3
Pathologic N		
NO	335	32.5
N1	95	9.2
N2	68	6.6
N3	2	< 0.1
Pathologic M		
MO	340	33.0
M1	25	4.86
Mx	144	14.0
Vital status		
Alive	333	32.3
Dead	181	17.6

### Annotation analyses of the DE lncRNAs and mRNAs

**Fig. 3:**
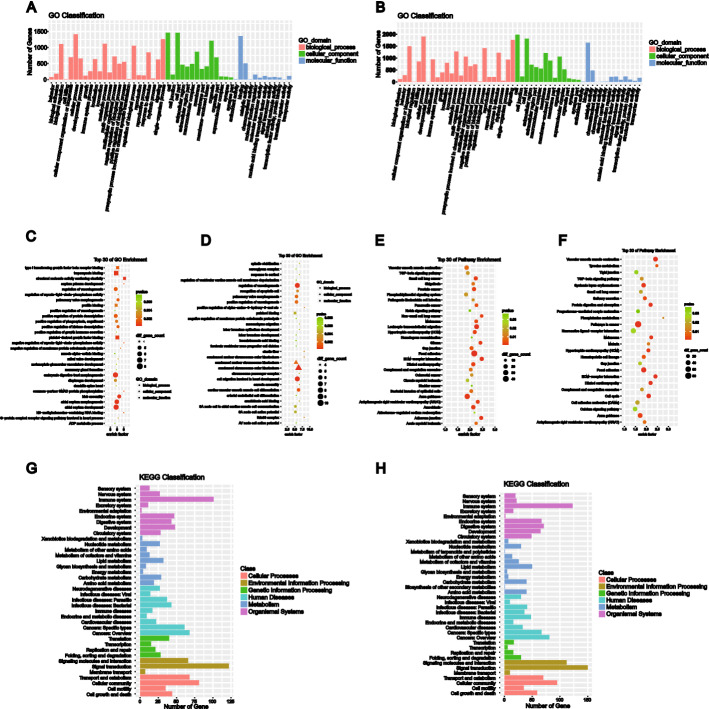
GO and KEGG enrichment analysis of DE lncRNAs and mRNAs. Barplot of the top 30 enriched GO classification terms for DE lncRNAs (**A**) and DE mRNAs (**B**). Bubble plot of the top 30 GO level 2 terms enriched by the DE lncRNAs (**C**) and DE mRNAs (**D**). Bubble plot of the top 30 KEGG pathways enriched by the DE lncRNAs (**E**) and DE mRNAs (**F**). **G**, **H** Barplot of the top 30 KEGG classifications for the DE lncRNAs (**G**) and DE mRNAs (**H**). GeneRatio ≥ 2, *P* < 0.05

GO analysis was used to annotate gene functions and standardize the descriptions of the DE genes according to the biological process (BP), cellular component (CC), and molecular function (MF) categories. We analyzed the cis-regulated lncRNAs and found that most of the top 30 GO terms enriched by the upregulated and downregulated genes (i.e., DE lncRNAs and mRNAs) were in the BP and CC categories (Fig. 3A, B). The top three descriptive terms enriched by the DE lncRNAs were atomic septum development, structural molecule activity conferring elasticity, and embryonic digestive tract morphogenesis (Fig. 3C). However, condensed chromosome outer kinetochore, cell migration involved in heart development, and regulation of vasculogenesis were the top three descriptive terms enriched by the DE mRNAs (Fig. 3D). Moreover, all the DE lncRNAs and mRNAs were involved in angiogenesis and cell proliferation.

The KEGG database, which enables pathway analysis of DE genes to identify biological functions, is divided into the following six classifications: cellular processing, environmental information processing, genetic information processing, human diseases, metabolism, and organismal systems. Comprehensive analysis of the KEGG classification results for the DE lncRNAs (Fig. 3G) and DE mRNAs (Fig. 3H) showed enrichment mainly in the terms signal transduction, immune system, and cancer: overview. Moreover, KEGG pathway enrichment analysis suggested that the DE genes were enriched mainly in vascular smooth muscle contraction, focal adhesion, and the TGF beta signaling pathway (Fig. 3E, F). Further analysis of the KEGG pathway term human diseases showed that these DE genes were closely related to small-cell lung cancer, NSCLC, melanoma, glioma, prostate cancer, thyroid cancer, colorectal cancer (CRC), and other tumors (Fig. 3G, H).

### Analysis of lncRNA target genes

**Fig. 4:**
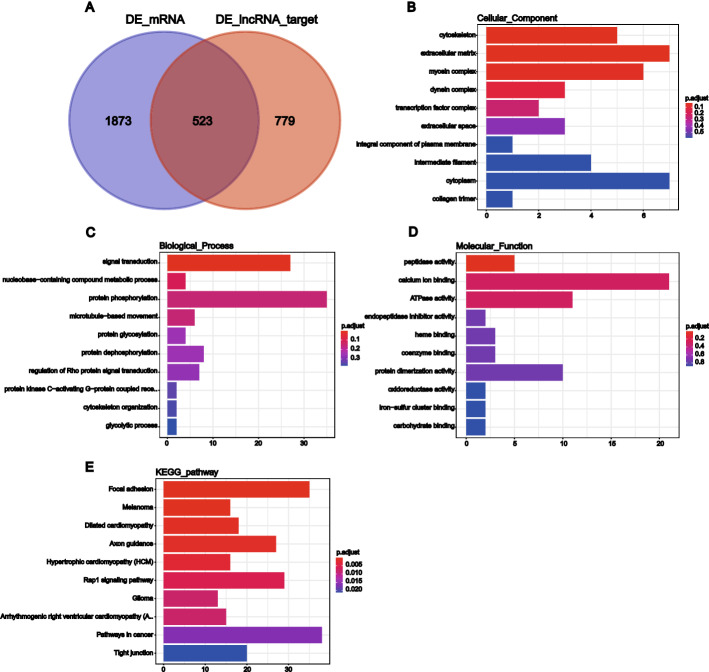
GO and KEGG enrichment analysis of the intersection between DE lncRNA target genes and DE mRNAs. **A** Venn diagram of DE mRNAs and DE lncRNA target genes. **B** Cell component (CC). **C** Biological process (BP). **D** Molecular function (MF). **E** KEGG pathway. The *y*‐axis shows significantly enriched categories for the targets, and the *x* axis shows the enrichment scores of these terms. The bar plot height indicates the number of genes in the functional area

To further clarify the functional annotations of the DE genes, we determined the intersection of the target genes of the 1302 DE lncRNAs and the 2396 DE mRNAs, and the resulting 523 common DE genes were selected with Venn diagram software (Fig. 4A). GO analysis showed that these genes were also enriched in the CC and BP categories. Moreover, the main enriched terms were extracellular matrix, myosin complex, and cytoskeleton in the CC category (Fig. 4B); signal transduction in the BP category (Fig. 4C); and peptidase activity in the MF category (Fig. 4D). The KEGG analysis results showed that these genes were enriched mainly in the pathways focal adhesion, axon guidance, differentiated cardiomyopathy, and melanoma (Fig. 4E). These results suggested that the identified DE genes may play important roles in cell morphology, adhesion, intercellular connections, and signal transduction.

### Candidate DE lncRNA validation in LUAD cell lines and OS analysis

**Fig. 5:**
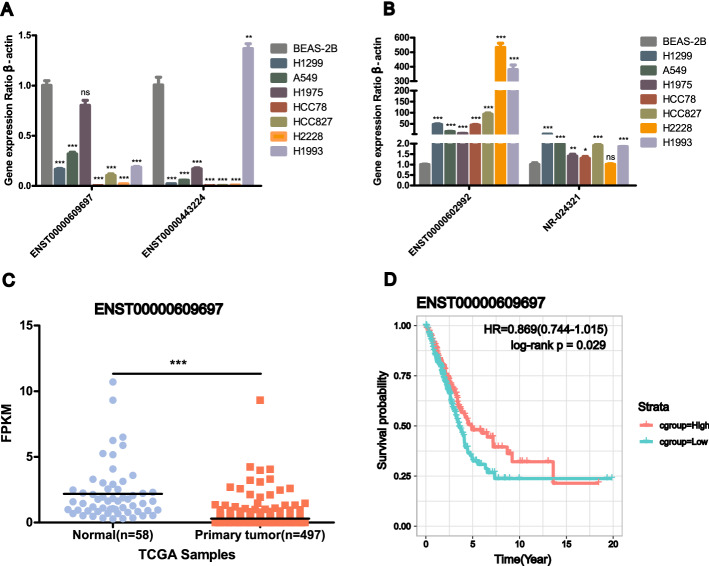
Validation of candidate lncRNAs. **A** The expression of the downregulation candidate lncRNAs in 7 LUAD cell lines and BEAS-2B cells was determined by qRT-PCR. **B** The expression of upregulation candidate lncRNAs in 7 LUAD cell lines and BEAS-2B cells was determined by qRT-PCR. The data are presented as the mean ± standard error of three independent experiments. *P < 0.05; **P < 0.01; ***P < 0.001. **C** The relative expression of the candidate DE lncRNA ENST00000609697 in the TCGA dataset. **D** Kaplan–Meier (KM) survival analysis based on the candidate DE lncRNA ENST00000609697. *x* axis: overall survival (years); *y* axis: survival rate. Green and red represent the low and high lncRNA expression groups, respectively

We selected four candidate DE lncRNAs from the 255 overlapping genes: two downregulated genes (ENST00000609697 and ENST00000443224) and two upregulated genes (ENST00000602992 and NR_024321). To confirm the screening results, the expression of the 4 DE lncRNAs was validated in 7 LUAD cell lines and compared with that in the BEAS-2B cell line using qRT-PCR (Fig. 5A). The expression of ENST00000609697 and ENST00000443224 showed a significant decreasing trend in almost all the LUAD cell lines, consistent with the microarray data (*P* < 0.05), while ENST00000443224 was upregulated in H1993 cells (*P* < 0.05) (Fig. 5A). The significant increasing trend (*P* < 0.05) in ENST00000602992 and NR_024321 expression was also consistent with the microarray data, but the increasing trend in NR_024321 expression was not obvious in H2228 cells (Fig. 5B). Next, we downloaded gene expression data and patient follow‐up data from the TCGA dataset to elucidate whether these candidate genes are potential prognostic markers for LUAD. Through TCGA dataset analysis, we found that ENST00000609697 was downregulated (*P* < 0.001) (Fig. 5C) and was the only candidate gene related to the prognosis of LUAD (log-rank *P* = 0.029) (Fig. 5D). ENST00000602992 and NR_024321 were upregulated in the TCGA dataset (*P* < 0.001) (Additional file 2: Fig. S1A, B). However, ENST00000602992 was not associated with the prognosis of LUAD (*P* = 0.24) (Additional file 2: Fig. S1C), and NR_024321 upregulation was not positively correlated with good prognosis in LUAD (*P* = 0.018) (Additional file 2: Fig. S1D). However, the downregulation of ENST00000609697 was positively correlated with good prognosis in LUAD; and therefore, this gene was considered a candidate biomarker that may function as a tumor suppressor.

### CeRNA regulatory network involving the DE lncRNAs

**Fig. 6:**
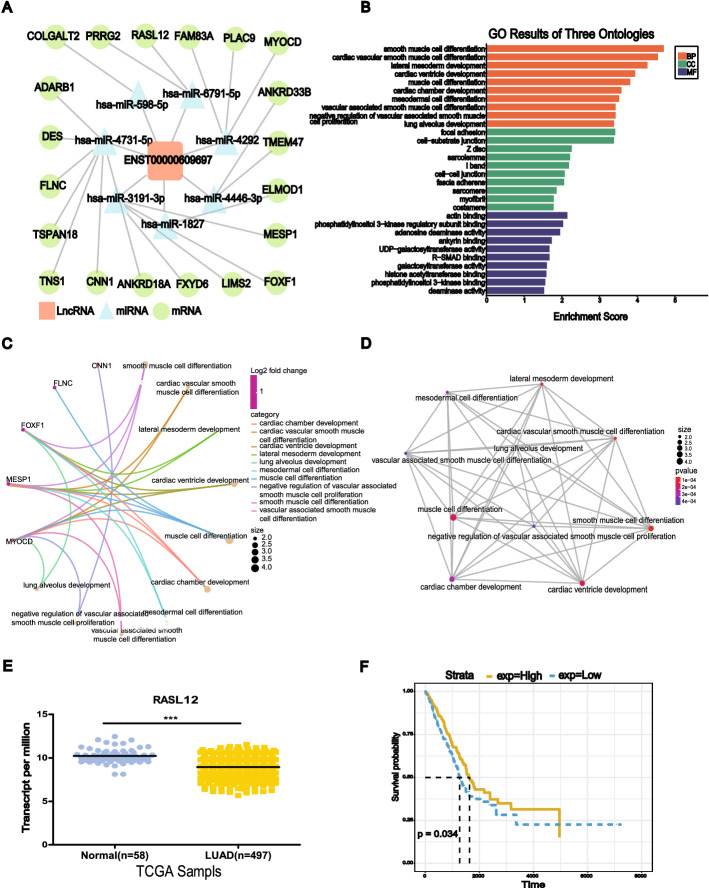
Target gene functional annotation of the ENST00000609697 ceRNA regulatory network. **A** CeRNA network of ENST00000609697. **B** Bar plot of the top 30 GO classification terms enriched by the target genes, *P* < 0.05. **C** BP cnetplot of the target genes, *P* < 0.001. **D** BP emapplot of the target genes, *P* < 0.001. **E** Relative expression of RASL12 in the TCGA dataset, *P* < 0.0001. **F** Survival analysis based on RASL12 in the TCGA dataset, *P* = 0.034

To further illustrate the potential interactions among the DE lncRNAs, miRNAs DE involved in LUAD, lncRNA–miRNA–mRNA ceRNA networks were constructed with a total of 188 DE lncRNAs, 444 DE miRNAs and 410 DE mRNAs (Additional file 2: Fig. S1E). Moreover, we found that most DE lncRNAs in the ceRNA regulatory network were downregulated. We selected the ceRNA network of the candidate gene ENST00000609697 for further analysis and found that it included 7 miRNAs (hsa-miR-3191-3p, hsa-miR-4731-5p, hsa-miR-598-5p, hsa-miR-6791-5p, hsa-miR-4292, hsa-miR-4446-3p, and hsa-miR-1827) and 20 DE mRNAs (COLGALT2, MYOCD, TNS1, RASL12, CNN1, etc.) (Fig. 6A). The enriched miRNA hsa-miR-4731-5p targeted most DE mRNAs in the ENST00000609697 ceRNA network, indicating that it may play a critical role in LUAD.

### Functional and survival analyses considering the target DE mRNAs in the ENST00000609697 ceRNA network

We next conducted GO enrichment analysis of the 20 targeted DE mRNAs in three ontologies: BP, CC, and MF. The 30 GO terms most enriched by the 20 targeted DE mRNAs are shown in Fig. 6B. The most enriched GO terms in the BP, CC, and MF categories were smooth muscle cell differentiation, focal adhesion, and actin binding, respectively (Fig. 6B). Most DE mRNAs mapped to the BP category; thus, we generated a BP cnetplot that showed the DE mRNAs associated with the top 10 BP terms; this analysis identified, 4 DE mRNAs (CNN1, FLNC, FOXF1, and MYOCD) (Fig. 6C). FOXF1 and MYOCD are related to multiple biological processes, suggesting that they may be critical genes in LUAD. The BP emapplot showed the overlapping relationship between each pair of terms (Fig. 6D) and suggested that smooth muscle cell differentiation was a very important biological process. To screen ceRNA networks related to LUAD prognosis, we downloaded expression and survival data related to the 20 target DE mRNAs in the ENST00000609697 ceRNA network from the UCSC Xena database. RASL12 expression was downregulated in LUAD (*P* < 0.0001) (Fig. 6E), and this downregulation was positively correlated with good prognosis (*P* = 0.034) (Fig. 6F). These results suggested that the ENST00000609697–hsa-miR-6791-5p–RASL12 axis may play a tumor-suppressive role in LUAD.

## Discussion

Here, we identified 2819 DE lncRNAs and 2396 DE mRNAs, including 859 upregulated lncRNAs, 1960 downregulated lncRNAs, 757 upregulated mRNAs, and 1639 downregulated mRNAs. More genes were downregulated than upregulated, indicating that downregulated genes may play important roles in the biology of LUAD. To explore the potential mechanisms of the DE genes, we performed GO and KEGG analyses of the aberrantly expressed lncRNAs and mRNAs. GO analysis showed that the DE lncRNAs were enriched mainly in atomic septum development, structural molecule activity conferring elasticity, and embryonic digestive tract morphogenesis and that the DE mRNAs were enriched mainly in condensed chromosome outer kinetochore, cell migration involved in heart development, and regulation of vasculogenesis. However, all the DE lncRNAs DE mRNAs were involved in angiogenesis and cell proliferation. Abnormalities in these two processes are closely related to the occurrence and development of cancers [25, 26]. The KEGG classification results for the DE lncRNAs and mRNAs showed that they were enriched mainly in signal transduction, the immune system, and cancers. Moreover, KEGG pathway enrichment analysis suggested that these DE genes were enriched mainly in vascular smooth muscle contraction, focal adhesion, and the TGF beta signaling pathway, and were also closely related to small cell lung cancer, NSCLC, melanoma, glioma, prostate cancer, thyroid cancer, CRC, and other cancers. According to the previous study, the focal adhesion and the TGF beta signaling pathways play essential roles in cell proliferation, and dysregulation of these two pathways is closely associated with oncogenesis [27, 28]. In addition, to further verify whether the functional annotations of the DE lncRNAs and mRNAs were basically consistent, we used Venn diagram software to intersect the DE lncRNA target genes and DE mRNAs and found 523 overlapping genes, which were involved mainly in the extracellular matrix, myosin complex, cytoskeleton, and signal transduction, among other processes. Moreover, the KEGG analysis results showed that these genes were enriched mainly in the focal adhesion and melanoma pathways. The main functional annotations of the DE lncRNAs and mRNAs suggested that these genes may play important roles in cell morphology, adhesion, intercellular connections, and signal transduction and are highly related to cancer. Thus, they are worthy of further analysis and verification.

An increasing number of aberrantly expressed lncRNAs are being identified as novel key regulators of the development of multiple human cancers [29, 30]. Aberrantly expressed lncRNAs may serve as biomarkers or function as oncogenes or tumor suppressors [29]; however, most studies have focused on lncRNAs as oncogenes. For instance, Song et al. reported that the protein claudin-4 encoded by the CLDN4 gene was upregulated in gastric cancer and related to poor prognosis [31]. The expression levels of the lncRNAs CCAT1 and CCAT2 were found to be significantly increased in CRC, and both lncRNAs were significantly correlated with poor relapse-free survival (RFS) and OS; these lncRNAs could thus be used independently or jointly as important prognostic biomarkers in CRC [32]. In addition, lncTCF7 was found to be significantly overexpressed in liver tumor tissues and liver cancer stem cells (CSCs); this lncRNA can recruit the SWI/SNF complex to the TCF7 gene promoter to regulate its expression, thus activating the Wnt signaling pathway [33]. In our study, the intersection of the 2819 DE lncRNAs identified by microarray analysis with the 1916 DE lncRNAs identified by TCGA database analysis revealed 255 overlapping DE lncRNAs: 161 downregulated and 94 upregulated. Then, we selected 4 candidate DE lncRNAs—2 downregulated (ENST00000609697 and ENST00000443224) and 2 upregulated (ENST00000602992 and NR_024321)—for validation in 7 LUAD cell lines. The expression of ENST00000609697, ENST00000602992, and NR_024321 was consistent with the microarray data. However, analysis of the relative expression levels of the candidate genes and the associations of these genes with patient survival in the TCGA dataset revealed that ENST00000609697 was downregulated and was the only candidate gene positively correlated with good prognosis in LUAD. Therefore, we considered ENST00000609697 a candidate gene that may be a novel tumor suppressor.

Recent studies have revealed that lncRNAs can act as ceRNAsby competitively binding to miRNAs to form lncRNA–miRNA–mRNA ceRNA networks and in turn play a critical role in the diagnosis, prognosis, and treatment of cancer [18, 34]. For example, lncRNA-KRTAP5-AS1 and lncRNA-TUBB2A can competitively bind miR-596 and miR-3620-3p as ceRNAs to promote CLDN4 expression, enhance cell proliferation and invasion, and promote epithelial–mesenchymal transition (EMT) [31]. To identify the potential interactions among the DE lncRNAs, DE miRNAs, and DE mRNAs, we constructed lncRNA–miRNA–mRNA ceRNA networks involving a total of 188 DE lncRNAs, 444 DE miRNAs, and 410 DE mRNAs. Interestingly, most of the DE lncRNAs in the ceRNA regulatory network were downregulated. We then screened the ENST00000609697 ceRNA network, which was downregulated and positively correlated with good prognosis in LUAD. This network contained seven miRNAs (hsa-miR-3191-3p, hsa-miR-4731-5p, hsa-miR-598-5p, hsa-miR-6791-5p, hsa-miR-4292, hsa-miR-4446-3p, and hsa-miR-1827) and 20 DE mRNAs (COLGALT2, MYOCD, TNS1, RASL12, CNN1, etc.). We performed an in-depth analysis of the functions related to the ENST00000609697 ceRNA network and found that the most enriched GO terms in the BP, CC, and MF categories were smooth muscle cell differentiation, focal adhesion, and actin binding, respectively. Smooth muscle cell differentiation is very important for the stability and repair of the vascular system, and abnormalities in this biological process can directly or indirectly affect the growth, proliferation, and migration of tumor cells and the tumor immune microenvironment [35–38]. Focal adhesions are the center of cellular mechanical sensation and serve as bridges between integrin, the extracellular matrix and the cytoskeleton, which is correlated with the tumor microenvironment. Changes in signal transmission through focal adhesions of malignant cells are very important for tumor cell metastasis [38–40]. Actin binding-related proteins participate in the formation of the cytoskeleton and regulate cell adhesion and migration [41]. The proliferation, migration, and invasion of tumor cells are dependent on proteins related to angiogenesis, focal adhesions, and actin binding. Therefore, the ENST00000609697 ceRNA network may play an important role in the tumor microenvironment of LUAD, and its functions are worth further exploration.

Subsequently, we downloaded expression and survival data for the 20 target DE mRNAs in the ENST00000609697 ceRNA network from the UCSC Xena database and found that RASL12 expression was downregulated in LUAD (*P* < 0.0001) and was positively correlated with good prognosis (*P* = 0.034). RASL12, a member of the RAS-like GTPase family, is localized in the cytoplasm [42]. However, evidence that RASL12 functions as a small GTP-binding protein is lacking; In fact, studies have reported that RASL12 could be homologous to the RAS-like GTPases RERG, RASL11A, RASL11B, RASL10A and RASL10B, which play tumor-suppressive roles in human cancers [43–45]. In addition, a recent study reported that the tumor suppressor RASSF1 can form a complex with RASL12 and recruit RASL12 to microtubules [46]. When combining these findings with our results, we inferred that RASL12 may be a tumor suppressor and that the ENST00000609697–hsa-miR-6791-5p–RASL12 axis may play a tumor-suppressive role in LUAD. More experiments should be performed to verify the role and regulatory mechanism of this axis.

## Conclusion

Our study identified DE lncRNAs and mRNAs in LUAD tissue samples via microarray profiling and bioinformatics analysis approaches. Our results showed that downregulation of ENST00000609697 and its target gene RASL12 was associated with poor prognosis in LUAD. We identified a novel ceRNA network (ENST00000609697–hsa-miR-6791-5p–RASL12) that might play a tumor-suppressive role. These results might indicate potential molecular therapeutic targets and biomarkers for LUAD.

## Materials and methods

### Patient selection and tumor tissue collection

None of the patients with newly diagnosed LUAD received radiotherapy or chemotherapy before surgery. LUAD tissues—both tumor and paracancerous (> 5 cm from the tumor) tissues—were obtained from patients during thoracic surgery at the Affiliated Hospital of Hebei University. Thirty-four pairs of tissue samples were collected and pathologically confirmed. We selected four pairs of tissue samples for microarray screening: four tumor tissues (1-C, 5-C, 9-C, and 12-C) and four matched adjacent normal tissues (1-N, 5-N, 9-N, and 12-N). Patient characteristics are presented in Table 4, and the quality results of tissue RNA are present in  Additional file 3.

**Table 4:** Summary of patient characteristics

Sample NO	Age	Sex	Pathological diagnosis	Clinical staging	Pathologic staging
1-C	53	Female	LUAD	cT2aN0M0 IB	pT1bN1M0 IIB
5-C	71	Male	LUAD	cT2aN2M0 IIIA	pT2aN2M0 IIIA
9-C	56	Female	LUAD	cT1cN2M0 IIIA	pT1cN2M0 IIIA
12-C	62	Male	LUAD	cT2aN0M0 IB	pT1cN2M0 IIIA

All tissue samples were kept in liquid nitrogen prior to RNA extraction. We selected the four samples based on the time of sample collection, RNA quality, and sex ratio. The four samples were stored in liquid nitrogen for no more than 60 days, and the RNA quality was A1. The male to female ratio was 1:1. The study was approved by the Clinical Research Ethics Committee of the Affiliated Hospital of Hebei University, and all tissue samples were collected with written informed consent from the patients (Additional file 3).

### Cell culture

The human bronchial epithelial cell line BEAS-2B and NSCLC cell lines (H1299, A549, H1975, HCC78, HCC827, H2228 and H1993) were purchased from the Typical Culture Preservation Commission Cell Bank of the Chinese Academy of Sciences (Shanghai, China) and had no mycoplasma contamination. The BEAS-2B cell line was cultured in BEBM supplemented with bronchial epithelial cell growth factor (BEGM Kit, LONZA Corporation, USA). The NSCLC cell lines were cultured in RPMI 1640 medium (H1299, H1975, HCC78, HCC827, H2228, and H1993) or F12K medium (A549) supplemented with 10% fetal bovine serum and 1% penicillin–streptomycin at 37 °C in a humidified atmosphere containing 5% CO^2^.

### Total RNA extraction and quantitative real-time PCR (qRT-PCR)

The total RNA was extracted from cells and tissues using TRIzol reagent (Invitrogen, USA) according to the manufacturer’s instructions. First-strand cDNA was synthesized using a Revert Aid First Strand cDNA Synthesis Kit (Roche, USA). qRT-PCR analysis of lncRNAs was performed in an Applied Biosystems 7500 Fast Real-Time PCR system (Applied Biosystems, USA) using One-Step SYBR PrimeScript (Roche, USA) according to the manufacturer’s instructions. The primer sequences are shown in Table 5.

**Table 5:** Primers used for qRT-PCR

Gene name	Forward primer (5′–3′)	Reverse primer (5′–3′)
ENST00000608161	AGCGTGTTCTCAGGAGCAGG	CACAGTTGCACAGACGACAGT
ENST00000609941	GGACAAGTGCTCAGAATTGCAT	CTTTTACTTAAGAGAATCTTTGCGGG
ENST00000609697	TGTGCTGTGTCCATCACCGA	TGATGCATTTATTACATTCCCAAAGCC
ENST00000443224	AGTGAAACTGTTGTCATCCTTAGTT	AGACAGTTCTAAACCAGACAATGACA
ENST00000602992	GACGCAGGGTGGTAGGGAAA	GGCTTCCCAGAGACACAAGC
ENST00000450016	CACTGCACTCCAGCTTGGGA	TTAATTTTTACAACAGCTTCCGGGGGA
NR-024321	TGGCTTGTCTTCCATCGTCC	GCACGAGGGTTGTTACAGGA
lnc-CDH1-5:1	CGGTCGGGTATGAGGCACAT	GCGCTGTGTGCATGTTGTTTG
β-Actin	CTCCTTAATGTCACGCACGAT	CATGTACGTTGCTATCCAGGC

The expression levels of lncRNAs were normalized to those of β-actin, and the relative lncRNA expression levels were calculated by the 2^−ΔΔCt^ method.

### LncRNA microarray analysis

The total RNA was amplified and labeled with a Low Input Quick Amp Labeling Kit, One-Color (Agilent Technologies, USA, Cat. #5190-2305), following the manufacturer’s instructions. Labeled cRNA was purified with a RNeasy Mini Kit (QIAGEN, GmBH, Germany, Cat. #74106). Each slide was hybridized in a hybridization oven with 1.65 μg of Cy3-labeled cRNA using a Gene Expression Hybridization Kit (Agilent Technologies, USA, Cat. #5188-5242) according to the manufacturer’s instructions. RNA samples from each group were analyzed using the SBC human ceRNA array V1.0 (4 × 180 K, Shanghai Biotechnology Co. Ltd., Shanghai, China), which contains approximately 68,423 lncRNA and 18,853 mRNA probes. LncRNA and mRNA probes were designed based on the latest genome version (human/grch38 (hg38)) with a probe length of 60 nt covering the GENCODE v21, Ensembl, UCSC, NONCODE, LNCipedia, lncRNAdb, and deepBase databases. Please refer to product information for details. The GeoPlatform number was GPL26192. Slides were scanned with an Agilent Microarray Scanner (Agilent Technologies, USA, Cat. #G2565CA) with default settings: dye channel, green; scan resolution, 3 μm; PMT, 100%; and range, 20 bits. The data were extracted with Feature Extraction software 10.7 (Agilent Technologies, Santa Clara, CA, USA).

Raw data were normalized with the quantile algorithm in the Limma package in R. Significantly DE transcripts were screened by FC ≥ 2 or ≤ − 2 and *P* ≤ 0.05. The screening principles to identify the four candidate genes were as follows: (1) DE lncRNAs were sorted by FC; (2) the difference in signal intensity between one pair of tumor tissue and normal control tissue was greater than 7 (downregulated DE lncRNAs, normal > 7; upregulated DE lncRNAs, cancer > 7) to facilitate later detection and verification; (3) the DE lncRNAs were located on chromosomes other than chrX and chrY; (4) the DE lncRNAs were shorter than 2 kb; and (5) exonic sense lncRNAs were excluded.

### Gene Ontology (GO) and Kyoto Encyclopedia of Genes and Genomes (KEGG) analyses

We performed GO enrichment (http://www.geneontology.org/) and KEGG pathway analysis (https://www.kegg.jp/). GO and KEGG enrichment analyses were performed with the functions *enrichGO* and *enrichKEGG* in the R package clusterProfiler [47] with the following parameters: (1) the gene of interest was assigned the gene vector using the Entrez Gene ID for database annotation mapping; (2) the QrgDb object (annotation db) was set with org.Hs.eg.db, the default object for Homo sapiens; (3) the *P* value was calculated by a hypergeometric distribution:

$$P=1-\sum\limits_{i=0}^{m-1}\frac{\left(\genfrac{}{}{0pt}{}{M}{i}\right)\left(\genfrac{}{}{0pt}{}{N-M}{n-i}\right)}{\left(\genfrac{}{}{0pt}{}{N}{n}\right)} ,$$

where *N* is the number of genes in the background, *M* is the number of genes annotated in the background to the biological term, *n* indicates the length of the gene vector provided in parameter one, and *k* is the number of genes annotated to the corresponding biological term in set *n*; and 4) the *P* value was adjusted for multiple comparisons with the BH (Benjamini and Hochberg) method. The terms identified by this analysis were arranged in descending order according to the enrichment factor value, and the top 30 terms were considered.

### Construction of the regulatory network

We selected the top correlated mRNA/lncRNA pairs in the normal and cancer datasets based on the correlation threshold of the 99th percentile of the corresponding overall correlation distribution in both cases. Then, we built up two regression models:

$$\rho XY|Z=\frac{\rho XY-\rho XZ\rho ZY}{\sqrt{1-{\rho }_{XZ}^{2}}\sqrt{1-{\rho }_{ZY}^{2}}}$$

### Seed match analysis

A perfect match at positions 2 to 7 of the 5ʹ-end of the mature miRNA sequence (6-mer miRNA seed) is the minimal pairing requirement considered predictive for miRNA target recognition. We used seed match analysis to restrict the above selected lncRNA/miRNA/mRNA triplets to those in which both the lncRNA and mRNA have at least one perfect 6-mer seed match for the shared miRNA.

By integrating the statistical analysis and seed match analysis results, we built the miRNA-mediated interaction (MMI) networks for both normal and cancer tissues. The nodes in these networks represent mRNAs and lncRNAs with highly correlated expression profiles, while the edges represent the miRNAs that mediate the interactions. The regulatory network was visualized using the Cytoscape tool [48] (3.9.0). The configurations (node color, position, and size) were chosen manually.

### Statistical analysis

All data are presented as the mean ± standard error. Paired samples compared using a paired two-tailed Student’s *t* test. Multiple group comparisons were performed with an one-way ANOVA followed by Dunnett’s multiple comparison test. Statistical analyses were performed with GraphPad Prism 5 (GraphPad Software, USA). *P* values less than 0.05 were considered to indicate statistical significance.

## Supplementary Information

**Additional file 1.** 255 overlapping genes.

**Additional file 2: Figure S1.** (A) The relative expression of the candidate DE lncRNA ENST00000602992 in the TCGA dataset. (B) The relative expression of the candidate DE lncRNA NR_024321 in the TCGA dataset. (C) Kaplan–Meier (KM) survival analysis based on the candidate DE lncRNA ENST00000602992. (D) Kaplan–Meier (KM) survival analysis based on the candidate DE lncRNA NR_024321. *x* axis: overall survival (years); *y* axis: survival rate. Green and red represent the low and high DE lncRNA expression groups, respectively. (E) CeRNA and coexpression regulatory networks of DE lncRNAs. CeRNA network: green and red represent down- and upregulated lncRNAs, respectively; □ represents lncRNA; ○ represents mRNA; the line represents miRNA; and the size of the circle or square represents the ability of the gene to interact with other genes.

**Additional file 3.** Tissue RNA quality.

## Data Availability

The datasets analyzed in this study are available from the corresponding authors on request.

## Abbreviations

lncRNAs: Long noncoding RNAs
LUAD: Lung adenocarcinoma
ceRNA: Competing endogenous RNA
DE: Differentially expressed
GO: Gene Ontology
KEGG: Kyoto Encyclopedia of Genes and Genomes
KM: Kaplan–Meier
NSCLC: Nonsmall-cell lung cancer
PFS: Progression-free survival
OS: Overall survival
MREs: MicroRNA recognition elements
BP: Biological process
CC: Cellular component
MF: Molecular function
AML: Acute myeloid leukemia
CRC: Colorectal cancer
PCC: Pearson correlation coefficient
