# Supplementary figures and images for: Identification of a novel prognosis-associated ceRNA network in lung adenocarcinoma via bioinformatics analysis
Source: Biomed Eng Online. 2021 Nov 24;20:117. doi: 10.1186/s12938-021-00952-x (PMC8611860; doi:10.1186/s12938-021-00952-x)

**A****ENST00000602992**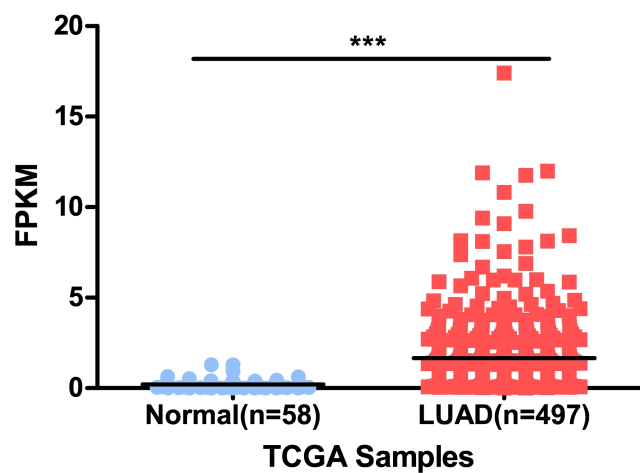**B****NR\_024321**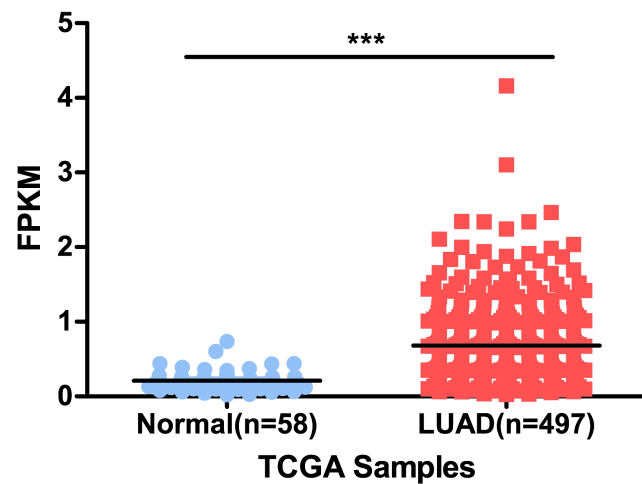**C****ENST00000602992**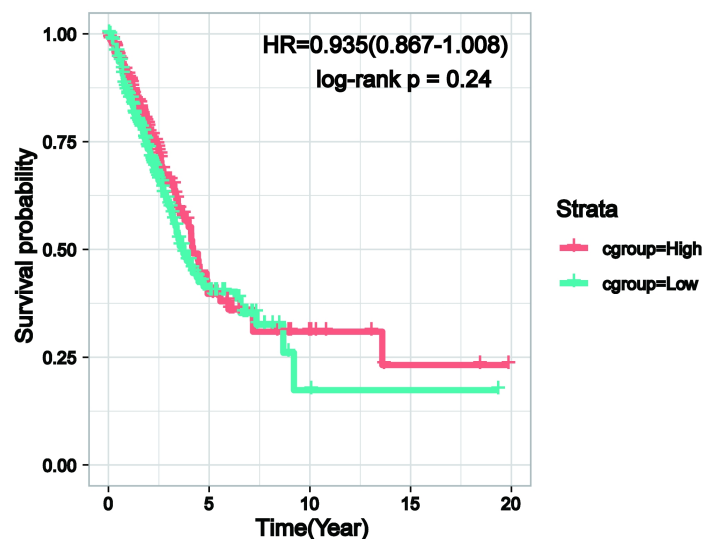**D****NR\_024321**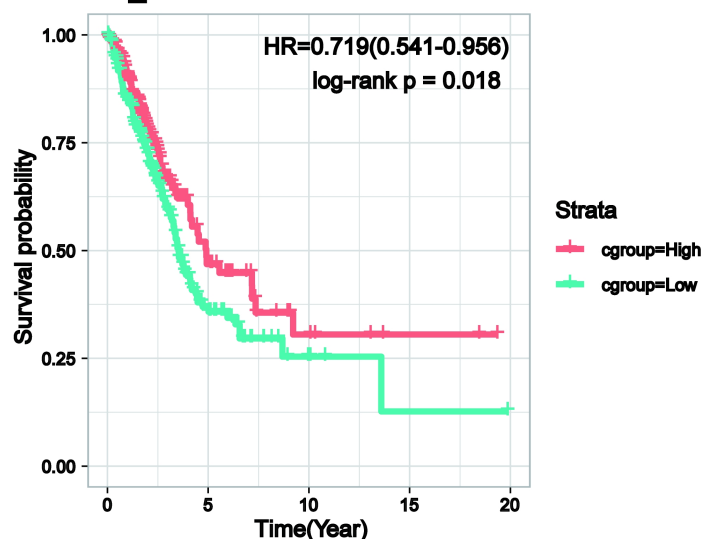**E**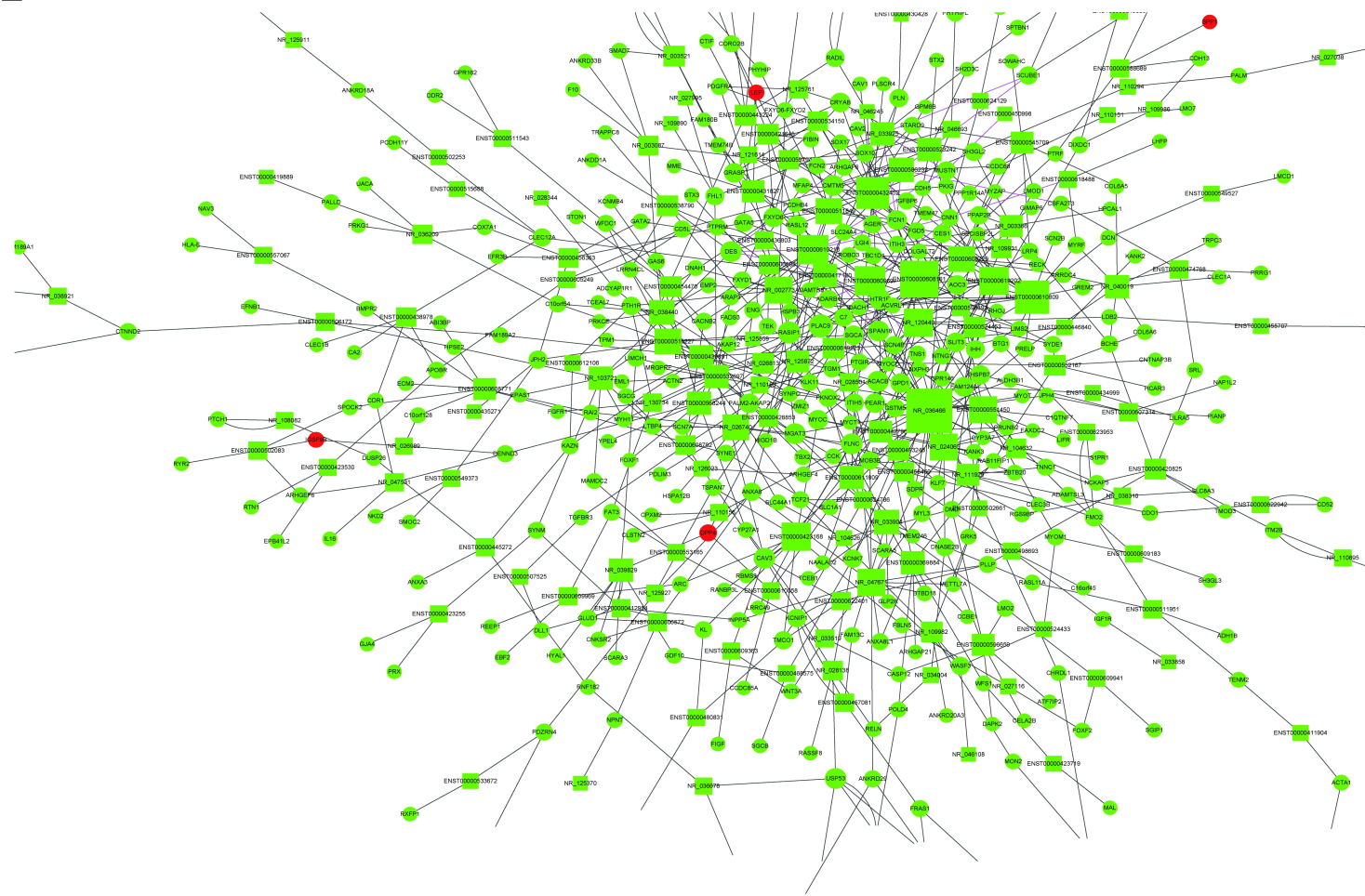

Supplement: Supplementary file 2 — Additional file 2: Figure S1. (A) The relative expression of the candidate DE lncRNA ENST00000602992 in the TCGA dataset. (B) The relative expression of the candidate DE lncRNA NR_024321 in the TCGA dataset. (C) Kaplan–Meier (KM) survival analysis based on the candidate DE lncRNA ENST00000602992. (D) Kaplan–Meier (KM) survival analysis based on the candidate DE lncRNA NR_024321. x axis: overall survival (years); y axis: survival rate. Green and red represent the low and high DE lncRNA expression groups, respectively. (E) CeRNA and coexpression regulatory networks of DE lncRNAs. CeRNA network: green and red represent down- and upregulated lncRNAs, respectively; □ represents lncRNA; ○ represents mRNA; the line represents miRNA; and the size of the circle or square represents the ability of the gene to interact with other genes. [file 12938_2021_952_MOESM2_ESM.pdf]
